# Avian Influenza at the Wild Bird–Poultry Interface: An Asia-Focused Review with Ecological Risk Scenarios for China

**DOI:** 10.3390/ani16131937

**Published:** 2026-06-23

**Authors:** Keyu Mo, Tingting Jiang, Peng Zeng, Yanli Zhong, Diqi Yang, Tingting Yu

**Affiliations:** 1 School of Animal Science and Veterinary Medicine, Hainan University, Sanya 572025, China; 2 Terrestrial Wildlife Rescue and Epidemic Diseases Surveillance Center of Guangxi, Nanning 530025, China

**Keywords:** avian influenza, highly pathogenic avian influenza, wild bird–poultry interface, migratory birds, domestic ducks, wetlands, environmental reservoirs, live poultry markets, ecological risk, China

## Abstract

Avian influenza remains a major threat to poultry production, wildlife conservation, and public health in Asia, where migratory birds, wetlands, rice paddies, domestic ducks, and live poultry trade often intersect. This Asia-focused review synthesizes ecological, epidemiological, surveillance, tracking, phylogenetic, and environmental evidence from 1996 to 2025, with particular emphasis on China, to clarify how risk develops at the wild bird–domestic poultry interface. The reviewed evidence suggests three broad epidemic phases: early Goose/Guangdong-lineage H5N1 outbreaks before 2014, recurrent clade 2.3.4.4 H5Nx expansions during 2014–2019, and the widespread clade 2.3.4.4b H5N1 period since 2020. Spatial risk is concentrated around major stopover wetlands and rice-paddy–duck landscapes, including Qinghai Lake, Poyang Lake, Sanmenxia, the Sanjiang Plain, and peri-urban market belts. Wetlands and paddies can maintain viruses environmentally, free-grazing ducks and bridge hosts can facilitate introduction, and live poultry markets and trade networks can amplify and export risk. By organizing these processes through an Interface–Amplifier–Conduit evidence-mapping approach, this review highlights setting-specific priorities, including seasonal wetland surveillance, closed farm-water systems, improved market hygiene, and better integration of ecological and genomic data for early warning and control.

## Simple Summary

Bird flu remains a major threat to poultry production, wildlife conservation, and public health, especially in Asia, where migratory birds, domestic ducks, farms, wetlands, rice paddies, and live poultry markets often overlap. This review explains how bird flu risk develops at the contact points between wild birds and domestic poultry, with particular attention to China. We summarize evidence from 1996 to 2025 and show that outbreaks are not driven by a single factor. Instead, risk increases when migratory birds gather in wetlands, domestic ducks or poultry share nearby water and feeding areas, and live poultry markets or trade routes help the virus spread further. Case examples from China, Bangladesh, and Japan show that different places have different dominant risks. Some are mainly shaped by shared water environments, while others are more influenced by dense poultry production and market movement. This review provides a practical way to compare these settings and highlights the need for stronger seasonal surveillance, safer farm water use, better market hygiene, and closer cooperation between wildlife, poultry, and public health sectors.

## 1. Introduction

Since the first isolation of highly pathogenic avian influenza virus (HPAIV) H5N1 from poultry in China in 1996, avian influenza viruses have continued to evolve and spread across Asia [1,2,3]. Hereafter, highly pathogenic avian influenza is abbreviated as HPAI and its causative viruses as HPAIV; low-pathogenic avian influenza viruses are denoted LPAIV. Early outbreaks caused by Goose/Guangdong (Gs/GD)-lineage H5N1 viruses were concentrated in East, South, and Southeast Asia before spreading westward to Europe and the Middle East, where localized epidemics occurred [4,5]. Following the emergence of clade 2.3.4.4 H5Nx viruses (i.e., H5 viruses bearing diverse neuraminidase subtypes, N1–N9) in 2014, this lineage rapidly expanded and triggered multiple intercontinental outbreaks [6,7]. Since the late 1990s, following the first reported human H5N1 outbreak in Hong Kong in 1997, avian influenza activity in Asia has displayed a persistent winter peak. Outbreaks typically surge from November to March, coinciding with the post-autumn congregation of migratory birds and intensified poultry trade and demand in winter markets, a pattern repeatedly supported by epidemiological studies [8,9,10,11]. Similar seasonal peaks and interface dynamics recur across Asian regions beyond China—e.g., Vietnam, Indonesia, and India—with local risk shaped by wetland configuration, poultry density, and market or trade connectivity.

This review synthesizes evidence on the spatiotemporal dynamics and risk factors of avian influenza at the wild bird–domestic poultry interface in Asia, with particular emphasis on China, from a bird-ecology perspective, to inform scenario-based monitoring and control. To organize heterogeneous evidence in a structured narrative synthesis, we used a descriptive Interface–Amplifier–Conduit (IAC) evidence-mapping approach. In this approach, interfaces denote ecological or production settings that create exposure opportunities among wild birds, domestic poultry, and contaminated environments, including wetlands, rice paddies, farm perimeters, shared water bodies, and live poultry market environments. Amplifiers denote hosts, facilities, or management conditions that may intensify local viral circulation after introduction, including dense poultry populations, mixed-species holdings, multi-source live poultry markets (LPMs), temporary holding facilities, and seasonal increases in poultry movement or demand. Conduits denote pathways that may facilitate wider spatial dissemination, including poultry trade, transport networks, migratory flyways, hydrological links, and farm–market movement chains. We do not propose a new theory or validated predictive model. Instead, the IAC approach was used descriptively to compare dominant exposure, amplification, and dissemination processes across ecological and production settings.

To apply the IAC approach consistently, evidence for each representative setting was mapped descriptively to one or more of the three IAC domains. Interface evidence included wetland proximity, rice-paddy–duck overlap, shared water use, wild-bird congregation, farm location, and seasonal migratory exposure. Amplifier evidence included domestic duck or chicken density, mixed-species holdings, LPM activity, market hygiene, local poultry management, and repeated viral detection or reassortment in dense poultry settings. Conduit evidence included poultry movement, trade connectivity, transport routes, market networks, flyway linkage, and spatial or genomic evidence suggesting wider spread. When multiple domains were supported, the dominant process was assigned according to the strongest and most convergent evidence available from surveillance, tracking, spatial, genomic, environmental, or intervention studies. Because evidence types and data resolution differed substantially among regions, these indicators were not combined into a formal numerical risk score. Instead, this approach was used to make qualitative comparisons of dominant risk processes and to organize setting-specific surveillance and control priorities.

## 2. Methods

### 2.1. Literature Search and Source Identification

This article was prepared as a narrative review with a structured evidence-mapping component. Literature searches covered studies and reports published or available online from 1996 to 2025. Main thematic searches were conducted in PubMed and Web of Science Core Collection using combinations of terms related to avian influenza, H5, wild birds, waterfowl, poultry, domestic ducks, live poultry markets, reassortment, environmental sampling, viral persistence, and Asian interface settings. Because this review also included representative regional case settings, targeted searches were conducted to identify additional studies related to Poyang Lake, the Sanjiang Plain, the Pearl River Delta, Bangladesh, and the Izumi Plain. The main thematic and targeted search terms and numbers of hits are summarized in Supplementary Table S1.

Official surveillance and guidance sources, including the World Organisation for Animal Health (WOAH), the Food and Agriculture Organization of the United Nations (FAO), and the European Food Safety Authority (EFSA), were searched separately to support sections on biosecurity, surveillance, outbreak control, and global control recommendations. Reference lists of relevant reviews and primary studies were also checked to identify additional publications.

### 2.2. Eligibility Criteria and Evidence Synthesis

Titles and abstracts were screened for relevance. Full texts were reviewed when records addressed one or more of the following topics: H5 highly pathogenic avian influenza (HPAI) occurrence or evolution; wild bird–poultry interfaces; domestic ducks and free-grazing duck systems; live poultry markets (LPMs); environmental detection or persistence of avian influenza viruses; reassortment or clade dynamics; flyway-linked dissemination; or biosecurity and outbreak-control measures. Records were excluded when they were unrelated to avian influenza, lacked relevance to animal, environmental, or interface processes, focused only on human clinical disease without animal or environmental context, or duplicated information available from more complete sources.

Records meeting these relevance criteria were retained for narrative synthesis and descriptive evidence mapping when they informed the focal interface types, representative case settings, or surveillance, biosecurity, and intervention evidence discussed in this review.

For each included source, information was extracted on geographical location, host species or environmental matrix, production or ecological setting, virus subtype or clade when available, study type, main findings, and relevance to exposure, amplification, dissemination, reassortment, environmental persistence, or control. Because this was a narrative review rather than a systematic review, evidence was not pooled quantitatively, and no formal meta-analysis was performed.

The extracted evidence was synthesized narratively and mapped to the Interface–Amplifier–Conduit (IAC) domains. In this approach, interfaces referred to ecological or production settings where wild birds, domestic poultry, bridge hosts, or contaminated environments could overlap; amplifiers referred to settings that could increase viral maintenance, mixing, or host density, such as LPMs, free-grazing duck systems, and dense poultry holdings; and conduits referred to mechanisms that could support wider dissemination, including migratory flyways, poultry trade, transport networks, and environmental connectivity. This mapping was used to compare dominant exposure, amplification, and dissemination processes across settings, rather than to build a formal predictive risk model.

### 2.3. Descriptive Outbreak Data Used for Figure 1

**Figure 1:**
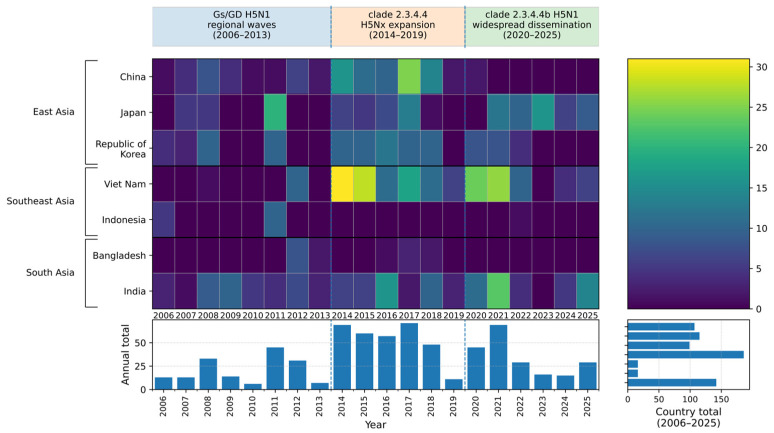
Annual H5 highly pathogenic avian influenza (HPAI) outbreaks in domestic poultry across selected Asian countries with major clade-phase annotations, 2006–2025. Rows represent countries grouped by Asian subregion; columns represent years; color intensity encodes the number of outbreaks per country–year. The top annotation band summarizes major lineage phases based on literature synthesis: Goose/Guangdong (Gs/GD)-lineage H5N1 regional waves (2006–2013), clade 2.3.4.4 H5Nx expansion (2014–2019), and widespread dissemination of clade 2.3.4.4b H5N1 (2020–2025). Vertical dashed lines mark the 2014 and 2020 phase boundaries. The lower bar chart shows the annual total number of outbreaks across the selected countries, and the right-hand bar chart shows cumulative country totals for 2006–2025. Outbreak counts were derived from the World Organisation for Animal Health (WOAH)–World Animal Health Information System (WAHIS) “Outbreaks” module, with disease = highly pathogenic avian influenza (H5), host = domestic poultry (non-wild birds), and Immediate Notifications and Follow-up Reports combined. Each outbreak was counted once per unique Reference and assigned to a calendar year using the Started-on date. Clade-phase annotations provide contextual lineage information and do not indicate clade-resolved counts within WAHIS. Data were extracted on 11 October 2025; counts for calendar year 2025 reflect reports available up to this date and may change as additional notifications are filed.

Descriptive outbreak data used for Figure 1 were obtained from the WOAH World Animal Health Information System (WAHIS) “Outbreaks” module and processed according to the following criteria: disease = highly pathogenic avian influenza (H5), host = domestic poultry (non-wild birds), report types = Immediate Notifications and Follow-up Reports, and countries = China, Japan, Republic of Korea, Viet Nam, Bangladesh, India, and Indonesia. Each outbreak was counted once per unique Reference and assigned to a calendar year using the reported “Started on” date. Data were extracted on 11 October 2025. These data were used only for descriptive visualization of country–year outbreak patterns and were not treated as clade-resolved counts or as a formal estimate of true incidence.

## 3. Spatiotemporal Dynamics at the Asian and Regional Scales

### 3.1. Asian Temporal Patterns

Avian influenza in Asia has followed a sustained but fluctuating evolutionary trajectory since the late 1990s. The highly pathogenic H5N1 virus of the Goose/Guangdong/96 lineage, first detected in Guangdong, China, in 1996, is widely regarded as the common ancestor of multiple Asian H5N1 lineages that continue to circulate, underpinning long-term transmission and phylogenetic diversification across Asia beyond the region [12,13,14].

Between 2003 and 2009, Gs/GD-lineage H5N1 strains—predominantly clades 2.2 and early 2.3.2—drove large-scale epidemic waves in Southeast Asia and subsequently spread to Europe and Africa [15]. By 2014, clade 2.3.4.4 H5Nx viruses (e.g., H5N6, H5N8) had emerged in China and other East Asian regions, establishing seasonal circulation with diverse transmission patterns [6,7,16]. From autumn 2020 onward, clade 2.3.4.4b H5N1 (a descendant subclade within clade 2.3.4.4 of the Gs/GD lineage) disseminated across continents through migratory bird movements, rapidly becoming the dominant global lineage and causing extensive epidemics in wild birds, domestic poultry, and several mammalian species, with subsequent incursions into North America, South America, and Antarctica [17,18,19]. Multiple datasets and reviews document widespread 2.3.4.4b-driven outbreaks across Asia during 2022–2023, with broadly synchronized winter peaks along major Eurasian flyways, while concurrent epidemics outside Asia indicate intercontinental circulation. During 2021–2022, avian influenza activity intensified along Asian flyways, with pronounced winter outbreaks in East Asia [20,21,22]. In 2022–2023, Japan experienced its largest recorded epidemic to date (84 poultry premises affected) [23], and the Republic of Korea reported 75 poultry-farm infections alongside 174 wild-bird detections [24,25]. In 2024, multiple H5 subtypes (H5N1, H5N2, H5N9, etc.) continued to be reported across Asia, with clade 2.3.4.4b remaining dominant, causing large-scale wild-bird mortality and sporadic spillover into mammals [18,26,27]. Meanwhile, earlier H5 lineages in China and neighboring regions (e.g., clade 2.3.2.1a/c/e and residual 2.3.4.4 H5N6) appear to persist at low levels alongside newer H5Nx viruses, sustaining opportunities for reassortment in domestic duck and poultry systems [14,16,28,29,30].

Based on the studies reviewed above, the epidemic history in Asia can be broadly divided into three periods: (i) ≤2013, outbreaks dominated by H5N1 (clades 2.2 and early 2.3.2) characterized by occasional regional waves; (ii) 2014–2019, repeated clade 2.3.4.4 H5Nx expansions across Asia with recurrent winter peaks; and (iii) 2020–present, dominant clade 2.3.4.4b H5N1 with repeated long-distance spread primarily mediated by migratory birds, pronounced winter peaks, and an expanding geographic footprint (Figure 1).

### 3.2. Asian Spatial Patterns: Flyways, Stopovers, and Recurrent Interface Landscapes

The East Asian–Australasian Flyway (EAAF) and Central Asian Flyway (CAF) are widely recognized as critical corridors for interregional spread of avian influenza in Asia [31,32]. These flyways operate through networks of breeding, stopover, and wintering sites where migratory birds aggregate and interact with local environments, thereby shaping when and where wild bird–poultry interface exposure occurs. Seasonal migration not only drives long-distance movement of H5 viruses [33], but the spatial configuration of stopover sites also exerts a significant influence on poultry outbreak risk. Tracking studies show that when farms fall within mapped wild-waterfowl habitat, outbreak risk increases by ~3–8-fold [34]. Regional surveillance further indicates that wetlands in Mongolia and Siberia serve as frequent sites of HPAI detection and may function as amplification nodes in cross-border transmission [35]. In Southeast Asia, mudflats and rice paddies (e.g., Thailand and Vietnam) provide shared habitats for migratory birds and domestic poultry, increasing HPAI transmission risk at these interfaces [36,37].

Satellite tracking and viral genomic evidence suggest that key EAAF stopover sites—such as Qinghai Lake, Poyang Lake, and Sanmenxia—operate as recurrent interface hubs. During seasonal congregation periods, elevated wild-bird abundance and environmental contamination around these sites are associated with higher spillover pressure and subsequent outbreaks in nearby poultry [11]. High-altitude migrants, including bar-headed geese, span routes from western China to South Asia and can act as cross-border hosts, potentially facilitating large-scale spread [38,39]. The 2005 H5N1 outbreak at Qinghai Lake, which killed over 6000 migratory birds, provided strong evidence that migration-linked congregation can contribute to regional and transboundary spread [40]. In 2015, H5N1 isolates from bar-headed geese at Qinghai Lake were highly similar to a swan goose strain from Sanmenxia [41], further supporting long-distance east–west linkage via migration.

Beyond China, several recurring interface landscapes shape H5 risk across Asia. In Vietnam, particularly in the Mekong Delta rice-paddy–duck production zone, free-grazing and itinerant duck flocks move between harvested paddies and wetlands, frequently overlapping with migratory waterfowl. Landscape-level risk is repeatedly associated with domestic-duck abundance and rice-cropping intensity, reflecting how paddies and flood cycles structure duck density and exposure opportunities [37,42,43,44]. In Indonesia (notably Java and Bali), LPMs form dense, highly connected retail and redistribution networks, where multiple clades/genotypes co-circulate and market connectivity sustains frequent detection and high genetic diversity over time [45,46,47]. In South Asia, including northeastern India and Bangladesh, regional risk mapping identifies domestic-duck density as the strongest predictor delineating high-risk areas, often coupled with wetland- and rice-based agroecological mosaics that support duck husbandry and interface exposure [48,49].

Stopover wetlands along Asian flyways concentrate large and diverse wild-bird assemblages, creating recurrent hubs where viruses can be maintained and redistributed through shared water bodies and fecal–oral transmission. When these aggregation sites lie adjacent to rice-paddy–duck landscapes or poultry production and marketing zones, short-term spikes in wild-bird abundance and environmental contamination can raise spillover pressure on nearby domestic flocks. Accordingly, priority stopover wetlands in the EAAF and CAF should receive intensified surveillance by combining routine wild-bird monitoring with targeted environmental water (and, where feasible, sediment) sampling during peak migration. Overall, seasonal migration and congregation patterns define the spatiotemporal window of wild-bird–poultry contact across Asia, with hotspots emerging where heavy wild-bird use of stopovers overlaps high poultry exposure in surrounding interface landscapes.

### 3.3. Epidemic Distribution in China’s Ecological Zones

China’s vast territory exhibits substantial ecological heterogeneity, resulting in marked variation in avian influenza risk across regions. Based on studies of H5 viruses (including recent clades up to 2025) and of H7N9 outbreaks since 2013, China shows clear seasonal and regional contrasts in outbreak distribution. During spring and summer, risk tends to be higher in wetland-rich regions of northern and western China, whereas winter risk concentrates in the eastern coastal plains and major lowland basins [50,51]. For example, spatial models integrating remote-sensing-derived water and land-cover variables with poultry distributions mapped high wild-bird–poultry interface risk in northeastern China (including the Sanjiang Plain) and the Yangtze River plain/Delta during the breeding season, whereas predicted risk shifted toward southern China and the middle–lower Yangtze River basin during winter [52]. Agroecological analyses further support surface water, rice agriculture, and domestic waterfowl density as key spatial correlates of H5 risk in China [53]. Global and regional risk mapping likewise highlights surface-water coverage and domestic duck/chicken densities as major predictors of H5 occurrence across Asia [37,53,54].

Within China’s migratory flyways, multiple monitoring studies indicate that the annual winter peak (November–February) in Anatidae abundance—including wild ducks and Baikal teal—coincides with H5 outbreaks in China [11,55]. This temporal overlap is consistent with the distribution of major wetland complexes and seasonal waterfowl aggregation areas nationwide, indicating that wetland location and bird use provide a spatial baseline on which poultry exposure amplifies risk. Overall, outbreak distribution in China reflects the joint effects of wetland resources and poultry production patterns: wetland complexes with high wild-bird densities and dispersed farms (e.g., Northeast China) show recurrent interface-driven outbreaks, whereas coastal plains with dense poultry and extensive market networks exhibit elevated amplification and spread risk.

## 4. Interface Transmission Mechanisms

### 4.1. Species Roles, Bridge Hosts, and Interface Ecology

Wild waterbirds, particularly Anseriformes and Charadriiformes, are recognized as the primary natural reservoirs of avian influenza viruses (AIVs) and have co-evolved with multiple subtypes over long periods. In these waterbird communities, AIV transmission occurs mainly via indirect fecal–oral or waterborne routes in shared wetlands, enabling sustained viral shedding without overt clinical signs and facilitating broad dissemination [56,57]. Ducks and geese can shed AIV during migration stopovers, continuously seeding the environment. Gulls and shorebirds frequent coastal wetlands and freshwater mudflats, where H5N1 and H9 have been detected in surveillance; they may also contribute to viral seeding along coastal flyways. Raptors (e.g., eagles, hawks) and scavengers (e.g., vultures) are usually infected after consuming infected carcasses or preying on debilitated birds, often developing severe disease or death, thus acting as indicators of HPAI circulation [58,59].

Beyond reservoir hosts, bridge hosts are synanthropic or opportunistic species that may facilitate virus movement between wild birds and poultry without serving as primary reservoirs. Synanthropic species near poultry operations (e.g., sparrows, pigeons, magpies, and other corvids) frequently enter farms, feed stores, and barns, and may therefore create opportunities for virus movement between wild-bird reservoirs, contaminated environments, and poultry flocks [59,60,61]. For example, experimental studies show that Eurasian tree sparrows can transmit H5N1 to co-housed chickens, with up to 100% mortality under specific caging conditions [62], whereas domestic pigeons show lower susceptibility and limited shedding, and are more likely to act as carriers [56]. Field investigations during outbreaks have repeatedly detected exposure or virus positivity in synanthropic birds around poultry premises, providing circumstantial support for their potential role as bridge hosts [59,61]. Scavenging species such as crows and vultures have also tested positive, likely due to feeding on infected carcasses, and may contribute to environmental dissemination where poultry and wild birds share landscapes. These findings represent different levels of evidence. Experimental studies demonstrate transmission capacity under controlled conditions, whereas field detections, genomic similarity, shared water use, and habitat overlap provide circumstantial or ecological support for suspected transmission pathways. Direct evidence of wild bird–poultry transmission requires a complete evidence chain linking infected wild or bridge hosts, contaminated environments, susceptible poultry, and the likely direction of transmission.

Importantly, host susceptibility to HPAI varies among species: ducks may tolerate H5N1 infection without apparent symptoms, whereas chickens and raptors often succumb—an asymmetry with direct implications for surveillance and risk assessment. Laboratory studies show that AIV persists longest in surface water at low temperature (<17 °C), neutral–slightly alkaline pH (about 7.0–8.5), and low salinity (<0.5 ppt), conditions typical of winter wetlands and irrigation waters that link wild waterfowl and domestic ducks [63]. Therefore, winter wetland and paddy water networks can act as high-persistence environmental interfaces that prolong indirect exposure; at these shared feed and water points, synanthropic bridge hosts may further carry viruses into poultry holdings.

### 4.2. Interface Types and Transmission Pathways

Poultry production and trade systems encompass several interface types with distinct transmission dynamics: (i) backyard and small-scale free-range systems; (ii) commercial large-scale production systems (caged or free-range); (iii) live poultry markets (aggregation, holding, and slaughter); and (iv) farm–wetland or shared-water overlap zones. Each interface presents distinct exposure risks, amplification mechanisms, and spread pathways.

In backyard and small-scale systems, poultry often roam free, or semi-free-ranging and overlap spatially with wild waterfowl in wetlands or rice paddies; geographic and epidemiological analyses have shown that the abundance of free-grazing domestic ducks is strongly associated with H5N1 outbreaks [64]. In Southeast Asia, spatial models likewise link H5N1 risk to duck abundance and rice-cropping intensity across Thailand and Vietnam [37]. Field sampling has detected evidence of exposure in synanthropic birds (e.g., house sparrows, starlings) at outbreak sites, suggesting that these species may be involved in local interface exposure or act as potential bridge hosts between contaminated environments and poultry flocks [61]. This interface is characterized mainly by environment-mediated fecal–oral transmission and indirect spread via contaminated drinking water and feed. Because these systems are widespread and closely tied to community livelihoods, they create a diffuse, persistent exposure network that is difficult to interrupt through centralized culling or single-point interventions.

Commercial poultry farms, while characterized by centralized management, remain vulnerable to introduction via multiple factors. Key determinants include farm location (e.g., proximity to wetlands), production systems (free-range or semi-open vs. caged), facility layout, and the rigor of biosecurity implementation. At the location scale, fine-resolution tracking and multilevel logistic regression found that poultry farms situated within mapped wild-waterfowl habitat had about 3–8-fold higher odds of an HPAI outbreak than farms outside such habitat [34]. Case–control studies reported that farms observing wild waterfowl or shorebirds in fields close to barns had roughly six-fold higher odds of HPAI incursion (odds ratio [OR] = 5.80, 95% confidence interval [CI]: 0.70–79.40; OR = 6.02, 95% CI: 1.83–19.78), whereas basic entry hygiene and personnel-control measures were consistently protective [65,66]. Thus, farm-level risk is shaped not only by scale but by interaction between management practices and the surrounding ecological context.

Live poultry markets constitute dense, multi-source hubs where poultry from diverse systems and regions are aggregated [67]. Within these markets, different poultry species are kept, traded, and slaughtered in confined spaces, facilitating multiple routes—direct contact, contamination by feces and body fluids, and aerosols—placing workers and the public at risk [68]. In practice, the epidemiological role of LPMs is reinforced by their structural and environmental characteristics: high bird turnover and crowding, mixing of multi-origin and multi-species consignments, and repeated human–bird and bird–bird contacts create intense local amplification, while hygiene infrastructure, waste accumulation, water use, and disinfection routines determine how long viruses persist in the market environment and how easily they spill over to farms and surrounding communities. Consistent with this mechanism, policy interventions in Hong Kong demonstrated measurable effects: banning overnight poultry storage combined with scheduled weekly rest-days and deep cleaning reduced influenza A virus isolation by about 84% in chickens and nearly 100% in minor poultry (e.g., quail) [69].

The interface between poultry farms and wetlands or other shared water bodies poses a distinct ecological risk. Wild waterfowl frequently rest/forage in wetlands or rice paddies, releasing virus into the water; sediments can act as long-term sources at low temperatures [70]. Meta-analyses confirm temperature as the dominant driver of persistence, with contributions from pH, salinity, and water type [71]. Consistent with these patterns, lab models indicate the longest persistence at <17 °C with pH 7–8.5 and low salinity, matching winter wetland conditions [63].

**Figure 2:**
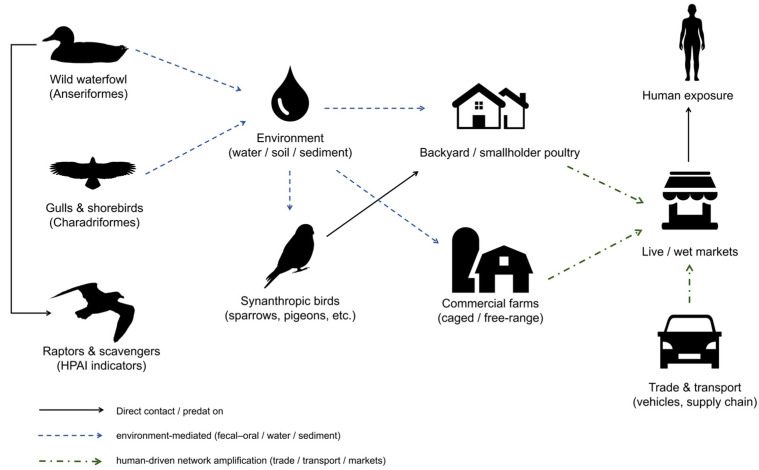
Ecological interfaces and transmission routes of avian influenza viruses (AIVs). Conceptual schematic of major interfaces and representative pathways linking wild birds, environmental reservoirs, poultry systems, markets, and human exposure. Arrows indicate hypothesized directions of movement or exposure, and node placement is illustrative rather than spatially scaled.

Based on the evidence reviewed above, three core mechanisms can be summarized: (i) direct-contact amplification (between species/within flocks, notably in markets and intensive farming); (ii) environment-mediated transmission (water, sediments, fomites—especially at backyard and wetland interfaces); and (iii) human-mediated spread (trade, transport, market hubs that extend local events regionally or cross-border). These mechanisms interact: market–transport hubs function as major amplifiers, while wetland–backyard interfaces act as persistent background sources of exposure (Figure 2).

Several practical priorities for risk reduction follow from the mechanisms outlined above. Reducing cross-exposure at high-amplification nodes is central, for example, by improving slaughter and circulation procedures and enforcing stricter transport disinfection. In wetland-adjacent areas, low-cost physical barriers such as enclosed water supply and night-time housing can provide added protection, while community-level biosecurity is most effective as a complement rather than a sole measure. Where farms lie within mapped wild-waterfowl habitat, the 3–8-fold higher odds of HPAI incursion justify site-selection buffers and closed-water systems as cost-effective priorities [34].

### 4.3. Mixed Farming and Reassortment Risks at Interfaces

Backyard, small-scale, and high-density poultry settings in which multiple poultry species or domestic waterfowl are kept in close proximity can create conditions for co-infection and reassortment [28,72]. These reassortment risks imply setting-specific surveillance and mitigation priorities. In commercial or high-density poultry farms, AIV detections should trigger subtype testing and, where possible, genomic sequencing to identify co-circulating subtypes or reassortants [72]. Farm-level mitigation should emphasize strengthened entry biosecurity, equipment and personnel hygiene, and measures that reduce introduction from wild birds or contaminated farm environments [65,66]. In LPMs, where dense market networks and live-poultry aggregation can support viral circulation, environmental and bird sampling should be paired with subtype monitoring [47,73,74]. If repeated positives are detected, mitigation can be escalated to limits on overnight poultry holding, scheduled rest-days, deep cleaning, temporary closure, and disinfection before reopening [68,69]. In small-scale, backyard, and free-grazing duck systems near wetlands or rice paddies, seasonal surveillance should prioritize domestic ducks, shared water sites, and mixed wild bird–duck interface areas, while mitigation can focus on reducing duck–wild-waterfowl overlap through measures such as protected or enclosed drinking-water systems, night housing during high-risk periods, and limiting access to open wetland habitats where feasible [64,68,75,76,77].

### 4.4. Market, Trade, and Farm Management: Human-Driven Mechanisms

Avian influenza is strongly shaped by human activities. Here, amplification mechanisms are considered across live poultry markets, poultry trade networks, and farm-level management practices. Live poultry markets, in particular, facilitate rapid viral amplification and cross-regional spread through crowding, species mixing, and frequent trade [73,78].

Beyond markets, poultry trade and transportation—including inter-provincial movement and illegal trade in wild birds—represent another critical component of the transmission chain. The structure of poultry trade networks strongly influences the spread of avian influenza viruses. For example, in Bangladesh, transmission models showed that observed farm–to–market spread could not be reproduced by assuming market-only transmission; incorporating poultry movement and trade links was necessary to explain the patterns, highlighting the key role of trade networks [67]. Similarly, in China, integration of viral genomic data with trade network analysis revealed that community structures within poultry trade networks were associated with localized viral transmission, further highlighting the importance of poultry trade in shaping epidemic dynamics [74]. These findings suggest that strict traceability, vehicle disinfection, and other biosecurity measures can help reduce transmission risk. Therefore, strengthening the regulation and biosecurity of the poultry trade and transportation is essential for controlling avian influenza spread.

In addition to markets and trade, farming practices play a decisive role in shaping the risk of avian influenza introduction and spread. At the farm management level, site selection and production systems critically determine exposure risks. Epidemiological and spatial analyses have shown that free-grazing systems, particularly free-ranging domestic ducks near wetlands or rice paddies, substantially increase the risk of HPAI introduction, as these farms spatially overlap with large numbers of migratory waterfowl during stopover seasons [37]. Moreover, field studies have identified a significant association between the presence of wild waterfowl or shorebirds around farms and subsequent viral incursions, and further highlighted the protective role of structured entry hygiene, such as requiring workers and visiting service crews to shower before barn entry (shower-in), change into farm-only clean clothing/boots after showering, and providing dedicated on-site restroom/portable toilet facilities for visitors to minimize contaminated re-entry [66]. Thus, the distinction between caged and free-ranging production is not merely a matter of farming style, but directly influences the frequency and intensity of wild-bird contact, and where farms lie within mapped wild-waterfowl habitat, fine-scale analyses report 3–8-fold higher odds, reinforcing the value of site-selection buffers and closed-water systems near wetlands [34]. Likewise, protective measures such as securing water sources, providing closed drinking systems, and ensuring strict sanitation and disinfection of personnel and equipment can substantially weaken wild bird–poultry interfaces, thereby reducing the probability of virus introduction and on-farm spread. Based on this evidence, farms located near high-risk areas—such as major wetlands or migratory stopover sites—should prioritize site-specific biosecurity measures, including protected or closed drinking-water systems, restricted access of free-ranging poultry to open wetlands, strengthened entry hygiene, equipment disinfection, and intensified surveillance during peak migration seasons.

## 5. Case Analysis of Typical Wild Bird–Domestic Poultry Interface in China and Asian Comparison

### 5.1. Case Analysis in China

In the Poyang Lake (Jiangxi) rice paddy–duck–wetland interface, the largest freshwater lake in China and a key wintering/stopover site on the East Asian–Australasian Flyway within the Yangtze River basin, hundreds of thousands of waterfowl congregate during the autumn–winter period. Field surveys and satellite telemetry in the Poyang Lake basin show strong spatial overlap among rice paddies, free-grazing domestic duck activities, and wild waterfowl habitats, supporting an environment-mediated exposure pathway through shared waters [55,77]. GPS loggers on free-grazing domestic ducks further demonstrate that domestic ducks frequently use paddies and adjacent natural wetlands also used by migratory ducks, with juvenile flocks roosting and foraging in close proximity during migration seasons, thereby elevating contact opportunities and potential onward dissemination along flyways [79]. More broadly, phylogeographic and spatial analyses across China and Southeast Asia link outbreak timing and distribution to local poultry density and management and rice–duck landscapes, supporting targeted hygiene, closed-water supply, and night-housing for smallholders [11,37].

In the Sanjiang Plain (Heilongjiang and Jilin), a rapidly expanding rice-paddy mosaic bordering natural wetlands, long-term reclamation and paddy expansion have transformed wetland margins, creating extensive shallow-water habitat that attracts Anseriformes during migration and wintering seasons. Remote-sensing and land-use analyses document rapid paddy expansion in northeastern wetland–farmland mosaics, including in the Sanjiang Plain, thereby generating extensive interface habitats where free-grazing domestic ducks and wild ducks share shallow-water habitats and foraging grounds. Telemetry and spatial epidemiology further highlight these overlap zones as recurrent contact hotspots [77,80]. Region-wide models in Asia identify domestic duck density and surface-water extent and wetland coverage among the strongest predictors of H5 risk, aligning with observations from the Sanjiang mosaic [49,76].

In the Pearl River Delta (Guangdong), peri-urban farms adjacent to rivers and ponds and embedded in dense market networks form a subtropical agro-aquatic landscape that interweaves concentrated poultry production with fish ponds and tidal flats, creating a typical farmland–wetland interface. Surveillance has repeatedly detected and isolated H5N6 viruses from wild waterfowl in Guangdong, confirming wild-bird carriage in the region; market and environmental genomic analyses reveal sustained H5N6 and H5N8 circulation and reassortment within LPM networks, indicating that the farm–market–environment chain acts as an amplifier–conduit complex [29,30,81]. Environmental and LPM studies in China regularly detect H5Nx in markets (e.g., positive rates around 8% for H5N6 in some LPM surveys), underscoring the value of scheduled market rest-days with deep cleaning as a high-leverage intervention [69,82].

### 5.2. Comparative Cases from Asia

In Bangladesh’s rice-paddy–wetland mosaics with nomadic and free-grazing ducks, wetland–agriculture systems create typical wild–domestic interfaces where these ducks commonly share winter feeding and resting sites with migratory waterfowl. Field surveillance in Bangladesh (2007–2012) has demonstrated widespread AIV detection in domestic ducks and LPMs, with multiple HA/NA combinations (including H5N1 and H9N2) circulating in ducks and market environments [47]. Spatial risk modeling implicates paddy distribution and domestic-duck abundance as significant predictors of outbreaks, highlighting the paddy–duck–market chain as the dominant pathway [83,84].

In Japan’s Izumi Plain (Kagoshima), a protected wintering site where cranes share roost waters with ducks, continuous monitoring has shown that H5N1 highly pathogenic avian influenza virus (HPAIV) and multiple low-pathogenic avian influenza viruses (LPAIV) subtypes can be repeatedly isolated from communal roost waters, underscoring the role of water as an environmental maintenance medium at wintering sites [85,86]. During targeted surveillance in the 2023–2024 winter, 45 H5N1 and 24 LPAIV strains were isolated from crane roost waters, with H5N1 detected first in four ducks in November and later in eight cranes in December. Phylogenetic analysis identified all isolates as belonging to clade 2.3.4.4b-G2d, and the early-winter duck and water isolates occupied more ancestral positions, supporting a transmission sequence in which migratory ducks likely introduced the virus, communal waters sustained it through environmental persistence, and cranes were subsequently exposed and infected [86]. In the same study, HI serology of six wintering cranes did not detect H5 HA–specific antibodies, suggesting that cranes are not the primary reservoir but rather spillover recipients that may contribute to local amplification within a transmission chain maintained by ducks and shared waters [87]. Taken together, the Izumi Plain observations from the 2022–2023 and 2023–2024 winters are consistent with an environment-mediated interface pathway, with migratory ducks acting as initial introducers and communal roost waters serving as a persistence medium that facilitates subsequent infection of cranes. Direct human influence appears relatively limited in this setting.

These comparisons suggest that interface risk varies substantially across settings (Table 1). Bangladesh is more strongly shaped by free-ranging domestic ducks in paddy–wetland systems, whereas the Izumi Plain case highlights waterborne maintenance after duck-mediated introduction. These differences indicate that control strategies should be adapted to the dominant processes in each setting.

### 5.3. Interface-Specific Transmission Patterns and IAC-Informed Intervention Priorities

Across lake/wetland interfaces (e.g., Poyang, Sanjiang, Izumi) characterized by high wild-waterfowl densities, free-ranging domestic ducks and farm poultry frequently contact wild birds directly or indirectly at shared water and feeding sites, creating conditions for virus circulation among wild birds, poultry, and the environment [55,87]. In rice-paddy–wetland systems (e.g., Northeast China and Bangladesh), shared water sources and virus deposition in paddies dominate, with nomadic duck flocks foraging alongside migratory birds and contributing to environmental viral load [88,89]. In coastal peri-urban zones (e.g., the Pearl River Delta), transmission is sustained mainly within dense local poultry populations; although direct wild-bird contact may be limited, proximity of farms to fishponds and irrigation channels allows contaminated water to serve as an entry and amplification route [81]. Across these settings, exposure is shaped mainly by wetlands and paddies, local amplification is strengthened by domestic ducks and live poultry markets, and wider spread is linked to trade and movement networks. These differences can be interpreted in relation to the three IAC domains described above.

Several practical priorities follow from these patterns. In wetland-adjacent areas, reducing direct and indirect contact between wild birds and poultry remains important, for example, by limiting access to open water and strengthening seasonal environmental monitoring. Farm-level measures such as sheltered rearing, closed drinking-water systems, and routine cleaning may further reduce environment-mediated exposure. In market-linked systems, rest-days, cleaning, traceability, and transport hygiene remain key components of control [47,90]. Where farms lie near major wetlands or mapped wild-waterfowl habitats, site-selection buffers and closed-water infrastructure are priorities; time-bounded surveillance surges should align with migration peaks to detect and respond early.

Using the IAC evidence-mapping approach described above, the three Chinese cases can be contrasted qualitatively. Poyang Lake is characterized by strong interface exposure and substantial local amplification, Sanjiang Plain is more clearly interface-dominant, and the Pearl River Delta is shaped more strongly by amplification and conduit processes related to dense production and market connectivity. This comparison is intended to illustrate relative differences in dominant exposure, amplification, and dissemination processes across settings. These interface-specific control and monitoring priorities are summarized in Table 2.

## 6. Control Challenges, Priority Evidence Gaps, and Intervention Priorities

### 6.1. Quantitative Evidence Summary Across Key Risk Drivers

Although most interface pathways remain qualitative, several studies provide repeatable quantitative signals that help prioritize surveillance and control. Key examples include: (i) fine-scale tracking and multilevel analyses reporting approximately 3–8-fold higher outbreak odds for poultry farms situated within mapped wild-waterfowl habitat than for farms outside such habitat [34]; (ii) a cross-sectional survey of backyard poultry farms around Poyang Lake quantified strong interface-related risks, with poultry sharing foraging space with wild birds showing OR = 6.57 (95% CI: 2.148–20.115), and purchase from live poultry markets showing OR = 3.74 (95% CI: 1.243–11.255) [75]; (iii) live poultry market interventions in Hong Kong reduced influenza A isolation by approximately 84% in chickens and nearly 100% in minor poultry after banning overnight storage and implementing scheduled rest-days with deep cleaning [69]; (iv) bridge-host experiments where Eurasian tree sparrows shed sufficient H5N1 to cause up to 100% mortality in co-housed chickens under caging conditions [62]; and (v) environmental persistence experiments indicating longest persistence in surface water at <17 °C, pH ≈ 7.0–8.5, and low salinity, parameters matching winter wetland and irrigation conditions linking wild-waterfowl and domestic ducks [63,71]. Together, these effect sizes are heterogeneous across settings but directionally consistent, supporting risk-stratified sampling and high-leverage hygiene actions at interface amplifiers. These quantitative signals are summarized in Table 3.

### 6.2. Limitations

This review is constrained by several limitations. First, surveillance and published datasets are biased toward poultry and LPMs, while wild-bird sampling and environmental sampling, including water, sediment, fecal, and market-surface sampling, remain sparse or intermittent across many flyways. Second, studies vary widely in scale, design, case definitions, and covariate control, which limits formal meta-analysis and leaves some pathways (e.g., bridge-host contact rates, wetland-distance thresholds, trade-network directionality) only weakly parameterized. Third, poultry production and trade data are often incomplete or aggregated, and genomic records may lack standardized interface metadata, reducing comparability across regions and years. Accordingly, the reliability of IAC-based comparisons depends on the availability and convergence of surveillance, tracking, spatial, genomic, environmental, and intervention evidence across settings. Our inferences should be interpreted as Asia-level, evidence-weighted tendencies; extrapolation to under-sampled flyways or interface types should be made cautiously until multi-year wild-bird and environmental datasets become available.

### 6.3. Research Gaps (Priority-Ranked)

(i) Surveillance remains biased toward poultry and LPMs, while repeated wild-bird and environmental monitoring across many Asian flyways is sparse or intermittent, limiting early-warning capacity at wetland–paddy interfaces.

(ii) Environmental persistence and operational interface thresholds (e.g., wetland-distance buffers, exposure-window length) are inconsistently quantified across ecological zones, constraining model transferability.

(iii) Bridge-host contact frequency and shedding parameters are rarely measured with standardized protocols, leaving a key interface pathway weakly parameterized.

(iv) Trade-network drivers are not routinely integrated with real-time genomic metadata, slowing traceback and cross-regional inference.

(v) Quantitative evaluations of interventions (especially LPM measures) are still limited across diverse Asian settings.

### 6.4. Priority Directions for Surveillance and Intervention

(i) Enhanced wetland surveillance during migratory periods.

At major stopover wetlands, surveillance may be intensified during periods of high migratory activity through repeated environmental sampling and, where feasible, targeted wild-bird sampling. Detection of H5 or H7 viruses in these areas should prompt closer monitoring in adjacent high-risk counties and may justify temporary reinforcement of local market hygiene and poultry movement control measures.

(ii) Adaptive hygiene cycles in live poultry markets.

In counties with elevated interface risk, live poultry markets may benefit from strengthened hygiene cycles during high-risk seasons, including regular rest days, deep cleaning, and restrictions on overnight poultry holding. Where environmental positivity persists across repeated rounds of surveillance despite routine hygiene cycles, responses may be escalated to temporary closure of affected LPMs, clearing of live poultry during closure, enhanced disinfection before reopening, and continued environmental sampling after reopening to monitor residual virus activity. Where recurrent market positivity persists, longer-term structural measures, such as shifting slaughter away from retail markets toward centralized processing, may also be considered according to local epidemiological and socioeconomic conditions [91,92,93,94].

(iii) Seasonal buffering at the farm–wetland interface.

For farms located close to wetlands or embedded in rice–duck systems, seasonal buffering measures may reduce opportunities for virus exchange between wild birds and domestic poultry. Relevant measures include enclosed drinking-water supply, night housing, and limiting access of free-grazing ducks to open wetland habitats. Farms with limited biosecurity capacity may be given priority in targeted surveillance and field supervision during migration peaks.

### 6.5. Global Biosecurity and Outbreak-Control Measures: Applicability to Asian Interface Settings

The intervention priorities outlined above are consistent with several globally recommended approaches for avian influenza prevention and control, but their applicability depends strongly on local production systems and interface types. Guidance from WOAH, the Food and Agriculture Organization of the United Nations (FAO), the FAO–WOAH global strategy, and the European Food Safety Authority (EFSA) emphasizes layered prevention, including farm biosecurity, separation of poultry from wild birds, protection of feed and water sources, surveillance and early reporting, cleaning and disinfection, movement control, zoning or compartmentalization, vaccination where appropriate, and outbreak-response measures such as targeted depopulation, compensation, safe carcass disposal, and post-outbreak disinfection [95,96,97,98,99]. In relation to the IAC organizing approach used here, these measures can be grouped into three levels: interface-level measures reduce exposure between wild birds, poultry, and contaminated environments; amplifier-level measures reduce viral persistence and multiplication in dense poultry or market settings; and conduit-level measures limit onward spread through poultry movement, trade, transport, or other connected pathways.

At the interface level, measures such as preventing contact between wild birds and poultry, keeping poultry indoors during high-risk periods, covering feed and water, separating ducks and geese from other poultry, and using closed or protected drinking-water systems are particularly relevant to commercial farms near wetlands, rivers, ponds, and rice-paddy landscapes [95,98,99]. In China, these measures may be feasible in commercial farms and peri-urban production systems where housing, water supply, and farm access can be managed. In contrast, year-round indoor housing is less applicable to free-grazing duck, nomadic duck, and smallholder systems in parts of Southeast and South Asia, where production is closely linked to harvested rice fields, seasonal flooding, and household livelihoods. In these settings, more realistic adaptations may include temporary housing during high-risk periods, night housing, reduced access to shared roosting waters during migration peaks, protected drinking-water points, and targeted monitoring of duck movements and environmental water.

At the amplifier and conduit levels, global recommendations overlap with the market- and movement-related measures discussed in Asian studies. In LPMs and temporary holding facilities, rest-days, market depopulation, prohibition of overnight poultry storage, cleaning and disinfection, species separation, reduced bird crowding, improved waste management, and traceability of poultry consignments may reduce repeated mixing and environmental persistence of avian influenza viruses [69,96]. These measures are relevant to regulated urban and peri-urban market chains, but their effectiveness depends on market infrastructure, trader compliance, and enforcement capacity. Movement control, transport disinfection, traceability, zoning, compartmentalization, and cross-sector data sharing are more readily implemented in integrated commercial systems than in informal poultry trade or nomadic duck systems [95,97]. Therefore, in smallholder-dominated settings, these measures may need to be combined with flock registration, mobile sampling along duck-grazing routes, temporary movement restriction during outbreaks, and coordination among veterinary, wildlife, market, and local administrative sectors.

Reducing unnecessary culling is also an important consideration in global avian influenza control. However, minimizing culling should not mean delaying outbreak response. Rather, it depends on earlier detection, targeted movement control, epidemiological investigation, safe carcass disposal, compensation mechanisms that encourage rapid reporting, and, where policy and surveillance capacity allow, vaccination combined with monitoring for virus circulation and antigenic change [95,97]. For China, risk-based control may be strengthened by combining farm biosecurity, LPM management, movement tracing, vaccination policy, and genomic surveillance. For Southeast Asian paddy–duck and smallholder systems, lower-cost and more flexible measures, such as seasonal buffering, temporary housing, safer water access, surveillance of free-grazing duck routes, and community-based reporting, may be more practical. Thus, global biosecurity recommendations are most useful when adapted to the dominant interface, amplifier, or conduit process in each setting rather than applied as a single uniform package.

### 6.6. Recent Evidence Reinforcing the Identified Gaps

Although evidence on wild bird–poultry interfaces continues to accumulate, key gaps remain in surveillance coverage, ecological mechanisms, and intervention effectiveness. First, spatiotemporal data on flyways and key stopover sites are still uneven in Asia. Recent surveillance syntheses and regional reports note that many secondary corridors and marginal sites lack consistent multi-year monitoring, which constrains fine-scale risk mapping and limits model reliability [100,101,102]. Second, virus persistence and transmission in environmental media require parameterized survival curves across ecological zones; recent environmental and laboratory-field studies reiterate that temperature, pH, salinity, and sediments strongly shape persistence and thus operational outbreak thresholds, highlighting the need for cross-zone calibration [103,104,105]. Third, field-level contact behaviors and the role of bridge hosts (e.g., synanthropic birds at feed and water points) remain insufficiently quantified with standardized contact matrices and shedding parameters, leaving evidence chains incomplete [59,106,107,108]. Finally, despite rapid progress in genomics and surveillance platforms, real-time cross-sector data sharing with harmonized metadata (host, precise location and time, interface type) remains inconsistent under a One Health framework, limiting early-warning and traceback [109,110].

## 7. Conclusions and Future Directions

Overall, the studies reviewed here suggest that avian influenza risk at wild bird–poultry interfaces in Asia is repeatedly shaped by the interaction of migratory waterfowl, shared wetland or paddy environments, domestic duck and poultry production, and connected market systems. Used descriptively, the IAC approach helps summarize how exposure, local amplification, and wider spread differ across settings.

This review synthesizes surveillance, tracking, phylogenetic, and environmental evidence, including virus detection in water, sediment, fecal, and market-environment samples, together with studies of viral persistence in environmental media under relevant temperature, pH, and salinity conditions, within a single comparative synthesis. Across the cases discussed here, several points recur: the importance of shared water environments, the role of domestic ducks and bridge hosts at interfaces, and the contribution of market and trade systems to wider dissemination. These observations may help guide more context-specific surveillance and control in China and other parts of Asia.

Future work should prioritize standardized, multi-year wild-bird and environmental surveillance along Asian flyways, harmonized metadata linking genomic records to host species, sampling matrices, and interface settings, and comparative evaluation of setting-specific interventions in market-linked, wetland-adjacent, and smallholder poultry systems.

## Figures and Tables

**Table 1:** Comparison of representative wild bird–poultry interface cases in Asia.

Region/Case	Interface Type	Seasonality	Key Hosts and Roles	Key Risk Features	Inferred Exposure Pathways	Potential Amplification
Poyang Lake (China)	Lake/wetland–paddy overlap	Autumn–winter	Migratory ducks (introducers); free-grazing domestic ducks (bridge/amplifier)	Strong paddy–duck–wetland overlap; shared water/foraging; high juvenile duck density	Environment-mediated exposure via shared waters; contact-associated duck–waterfowl overlap	Free-grazing domestic duck flocks; backyard/smallholder poultry
Sanjiang Plain (China)	Wetland–paddy mosaic (paddy expansion)	Migration and wintering	Migratory Anseriformes (exposure); domestic ducks (local amplifier)	Rapid paddy growth at wetland margins; shallow-water overlap hotspots	Environment-mediated exposure; contact-associated overlap in shallow-water habitats	Free-grazing domestic ducks; local poultry holdings
Pearl River Delta (China)	Coastal agro-wetland near ponds/canals	Year-round; spikes after water events	Dense poultry/ducks (primary amplifiers); wild waterfowl (carriage)	High farm/market density; water-network contamination; strong supply chain connectivity	Environment-mediated exposure; market- and supply chain-associated circulation	LPMs; transport/processing nodes; dense poultry holdings
Bangladesh	Paddy/wetland with nomadic ducks	Winter	Nomadic ducks (bridge/amplifier); migratory waterfowl (introducers)	Nomadic flocks connect sites; fecal shedding elevates paddy viral load; market co-detections	Environment-mediated paddy–duck exposure; market-associated circulation	Nomadic/free-grazing duck flocks; LPMs
Izumi Plain (Japan)	Shared roost-water wintering ground	Winter	Migratory ducks (introducers); roost waters (persistence); cranes (spillover)	Early duck detections → water maintenance → later crane cases	Environment-mediated exposure via communal roost waters	Communal roost waters; wild-bird aggregation

Inferred exposure/spread pathways and potential amplification or maintenance settings were summarized descriptively from the cited case studies. These categories indicate ecological, environmental, market-related, or movement-related processes suggested by the reviewed evidence, rather than formal evidence grades or direct proof of specific wild bird–poultry transmission events. LPM = live poultry market.

**Table 2:** Interface-specific risk, control, and monitoring priorities.

Interface Type	Dominant Risk Features	Priority Controls	Monitoring Focus
Inland wetland—paddy overlap	Wild ducks with free-grazing ducks; shared water and foraging areas	Seasonal buffering; penning or semi-penning of ducks; water quality; carcass removal	Sentinel flocks near wetlands; water sampling (roost/paddy); migration-season sweeps
Paddy/wetland with nomadic ducks	Nomadic flocks bridge sites; fecal–oral via shallow waters; multi-species contact	Rotational access; dry-down intervals; temporary holding away from shared roosts; hygiene at loading	Nomadic routes and stopovers; fecal/environmental samples; backyard clusters
Coastal agro-wetland near ponds/canals	Dense farms adjacent to ponds/canals; supply chain amplification; limited direct wild contact	Secure water intakes; farm–market segregation; LPM rules; vehicle/equipment sanitation	Intake water; market-chain nodes; high-density farms after rain/flooding

Environmental transmission refers to indirect transmission via water, sediments, or fomites. Amplification refers to rapid virus spread in live poultry markets or supply chain nodes. Abbreviation: LPM = live poultry market. These items are illustrative and should be adapted to local migration timing, farming systems, and hydrological conditions.

**Table 3:** Quantitative evidence supporting key interface risk drivers.

Theme/Risk Driver	Study (Year)	Metric Reported	Effect Size	Region/System	Notes
Wetland/wild-waterfowl habitat proximity to farms	Lee et al. (2020) [34]	OR (outbreak odds)	~3–8-fold higher odds	Poultry farms at the wild-bird habitat interface	Fine-scale tracking + multilevel logistic models
Backyard poultry contact with wild birds	Wang et al. (2013) [75]	OR (disease/death risk)	OR 6.573 (95% CI 2.148–20.115)	Backyard farms, Poyang Lake area, China	Wild-bird foraging overlap
Backyard purchase from local live bird markets	Wang et al. (2013) [75]	OR (disease/death risk)	OR 3.740 (95% CI 1.243–11.255)	Backyard farms, Poyang Lake area, China	Market-mediated introduction
LPM hygiene intervention (‘no overnight’ + rest days)	Leung et al. (2012) [69]	Percent reduction in AIV isolation	≈84% (chickens); ≈100% (minor poultry)	Retail LPMs, Hong Kong	Natural experiment/policy intervention
Bridge-host transmission capacity (sparrows to chickens)	Gutiérrez et al. (2011) [62]	Experimental mortality	Up to 100% mortality in co-housed chickens	Caging experiments	Controlled caging experiment; demonstrates transmission capacity under experimental conditions
Water persistence under winter wetland conditions	Keeler et al. (2014) [63]; Dalziel et al. (2016) [71]	Environmental conditions associated with prolonged AIV survival	Longest survival at <17 °C, pH 7–8.5, low salinity	Wetland/irrigation waters	Mechanistic support for environment-mediated interface

The quantitative metrics summarized in this table were extracted from heterogeneous study designs, including observational epidemiological studies, natural intervention studies, controlled transmission experiments, and laboratory or environmental persistence studies. Effect sizes are therefore not directly comparable across rows and should be interpreted as evidence supporting specific interface risk drivers rather than as pooled risk estimates. Approximate values are indicated with “~” or “≈” where exact values were summarized from the cited studies. Abbreviations: OR = odds ratio; CI = confidence interval; AIV = avian influenza virus; LPM = live poultry market.

## Data Availability

No new primary data were generated in this study. All evidence supporting this review is available from the cited literature and publicly accessible sources referenced in the manuscript. The descriptive outbreak data used for Figure 1 were derived from the WOAH WAHIS “Outbreaks” module using the criteria described in Section 2.3.

## Supplementary Materials

The following supporting information can be downloaded at: https://www.mdpi.com/article/10.3390/ani16131937/s1, Table S1. Main thematic and targeted search terms used for the narrative review.

## Abbreviations

The following abbreviations are used in this manuscript:

AIV	Avian influenza virus
CAF	Central Asian Flyway
CI	Confidence interval
EAAF	East Asian–Australasian Flyway
Gs/GD	Goose/Guangdong
HPAI	Highly pathogenic avian influenza
HPAIV	Highly pathogenic avian influenza virus
IAC	Interface–Amplifier–Conduit
LPAIV	Low-pathogenic avian influenza virus
LPM	Live poultry market
OR	Odds ratio
WAHIS	World Animal Health Information System
WOAH	World Organisation for Animal Health
EFSA	European Food Safety Authority
FAO	Food and Agriculture Organization of the United Nations
